# Not All Smokers Die Young: A Model for Hidden Heterogeneity within the Human Population

**DOI:** 10.1371/journal.pone.0087403

**Published:** 2014-02-10

**Authors:** Morgan Levine, Eileen Crimmins

**Affiliations:** Davis School of Gerontology, University of Southern California, Los Angeles, California, United States of America; National Jewish Health, United States of America

## Abstract

The ability of some individuals to reach extreme old age in the presence of clearly high exposure to damaging factors may signal an innate biological advantage. For this study we used data on 4,655 current and never smokers, ages 50 and above, from NHANES III to examine whether long-lived smokers represent a biologically resilient phenotype that could facilitate our understanding of heterogeneity in the aging process. Using a proportional hazards model, our results showed that while smoking significantly increased mortality in most age groups, it did not increase the mortality risk for those who were age 80 and over at baseline. Additionally when comparing the adjusted means of biomarkers between never and current smokers, we found that long-lived smokers (80+) had similar inflammation, HDL, and lung function levels to never smokers. Given that factors which allow some individuals to withstand smoking may also enable others to cope with everyday biological stressors, the investigation of long-lived smokers may eventually allow us to identify molecular and genetic mechanisms which enable longevity extension.

## Introduction

The rate of aging and subsequent mortality risk is hypothesized to result from the balance between the body's exposure to harmful environmental factors, and its genetically-determined ability to repair and protect against damage [1]. Thus, the ability of some individuals to reach extreme old age, particularly in the presence of clearly high exposure to damaging factors, may signal an innate resiliency that could be related to slower rates of aging. Genetic and environmental factors impact the rate of aging via a number of downstream physiological processes, for example: inflammation, oxidative stress, the accumulation of advantaged glycation end products that contribute to the cross-linking of proteins, loss of homeostatic control, and damage to DNA [2]–[4]. Cigarette smoking has been identified as an environmental factor with the ability to exacerbate a number of these processes [5], [6] and as a result, smoking has been associated with accelerated rates of physiological decline, increased disease incidence, and reductions in life expectancy [7], [8]. Nevertheless, some smokers do survive to extreme ages and these individuals may provide an opportunity to examine a resilient subgroup of the population and uncover the factors that impact susceptibility to physiological stressors.

Harman's free-radical theory of aging proposes that exposure to reactive oxygen species (ROS) is one of the major contributors to aging, and has been linked to increased risk of diseases such as cancer, cardiovascular disease, diabetes, and dementia [9]. Nevertheless, there is evidence of variation in the susceptibility to such damage. Studies on animal models suggest that longer-lived animals may possess innate stress resistance mechanisms allowing them to limit the amount of oxidative damage [10]. Additionally, oxidative stress associated inflammatory responses to endogenous and exogenous stressors may also contribute to differences in lifespan given its implications for the accumulation of cellular damage. Consequently, variations in innate immune response, either as a result of genetic or epigenetic factors, may have the potential to influence the aging process, to the degree that individuals with diminished pro-inflammatory activation may experience increases in longevity. Links between longevity and inflammation associated cellular damage are consistent with Kirkwood's disposable soma theory, which suggests that increased energy allocation towards physiological processes involved in somatic maintenance and repair, and away from those involved in growth and reproduction, contribute to life extension [11]. Therefore, given that smoking is associated with increased ROS exposure, and pro-inflammatory cytokine activation, individuals with genotypes associated with down regulation of inflammatory processes, and the up regulation of processes associated with cellular protection and regeneration may be less prone to the negative effects of cigarette exposure, thus enabling them to survive longer than other smokers.

Evidence of longevity associated resiliency to stressors has recently been documented in studies of centenarians [12], [13]. Results from these studies suggest that protection from oxidative stress and decreased production of pro-inflammatory cytokines may promote longevity in humans. Studies have also found significantly higher levels of high density lipoprotein cholesterol (HDL) among centenarian offspring compared to age-matched controls [14]. High-density lipoproteins (HDLs) have been shown to have antioxidant and anti-inflammatory properties and are associated with survival in late-life [15], [16]. Finally, although HDL levels are often reduced by smoking [17]—presumably contributing to increased risks for atherosclerosis—individuals with predisposed resiliency may not experience these declines.

Because the prevalence of individuals with high levels of resiliency may be small, differences in vulnerability to physiological stressors may be hard to detect in younger populations. This results from hidden heterogeneity, which refers to variability in the susceptibility to death within a population [18]. For a younger population which includes a large number of non-resilient individuals, the overall mortality risk will be representative of the general, non-resilient, sub-population [19]. However, as the frailer (more susceptible) individuals are selected out of the population via mortality, the resilient individuals begin to make up a larger proportion of the population, and the risk estimates for the group will start to resemble those of the resilient sub-population. Consequently, mortality selection may provide a convenient way to visualize hidden heterogeneity. While at younger ages we would expect smokers to have much higher physiological dysregulation and mortality than non-smokers—given that most of the smoking population is non-resilient—when comparing older smokers to non-smokers mortality should have already selected out the individuals that are not resilient to smoking, and as a result, the smokers who remain should be less susceptible to the negative effects of cigarette exposure.

Although the adverse effects of smoking on health have been well documented, little is known about whether individuals vary in their vulnerability to biological stressors, such as smoking. Using data from the National Health and Nutrition Examination Survey (NHANES III), this study aims to uncover 1) whether differences in mortality and levels of physiological dysregulation of smokers and non-smokers converge with age—signifying greater resilience among long-lived smokers, and 2) whether indicators of physiological dysregulation can be used to uncover hidden heterogeneity among smokers.

## Materials and Methods

### Study Population

The study was based on data from the third National Health and Nutrition Examination Survey (NHANES III), and included 4,655 adults ages 50 and over. Excluded subjects (n = 850) were those who reported past (but not current) smoking, and those with missing biomarker data. NHANES III is a nationally representative, cross-sectional study conducted by the National Center for Health Statistics (NCHS) between 1988 and 1994. Data for NHANES III were collected during at-home interviews, and physician examinations, which took place in a Mobile Examination Center (MEC). Biomarker, smoking status, and sociodemographic data were available for a single time-point when a participant was interviewed between 1988 and 1994. However, mortality follow-up was available for all participants through 2006. Further details on recruitment, procedures and study design are available through the Centers for Disease Control and Prevention [20].

### Smoking History

In order to test whether individuals chronically exposed to biological stressors, but surviving into extreme old age are more resilient, only two groups were compared—never-smokers and current smokers. Those reporting smoking in the past were excluded given the evidence that some of the negative effects of smoking can be reversed after cessation [21]. Persons reporting not having smoked at least one-hundred cigarettes in their lifetime were classified as never-smokers; while persons who report smoking at the time of interview were classified as current smokers. In addition, years of cigarette use and average number of cigarettes smoked per day were calculated for current smokers. The number of years of smoking was estimated as the difference between the age at which the subject started smoking and his/her current age. Periods of nonsmoking are also reported and any period of time during which subjects reported cessation were subtracted.

Daily smoking quantity was calculated based on smokers' answers to five questions—1) “About how many cigarettes do you smoke per day?”; 2) “For approximately how many years have you smoked this amount?”; 3) “Was there ever a period of a year or more when you smoked more than (number previously reported) cigarettes per day?”; 4) “During the period when you were smoking the most, about how many cigarettes per day did you usually smoke?”; and 5) “For how many years did you smoke that amount?”. Given that smoking patterns tend to change over the lifetime, both current and highest smoking rate was used to calculate average reported cigarette use. This was estimated by summing the number of cigarettes currently smoked per day (multiplied by the number of years smoking that quantity) and the number of cigarettes smoked per day at its highest (multiplied by the number of years smoking that quantity) and then dividing by the total number of years reported on.
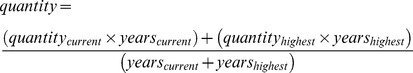



A variable for heavy smoking was created based on whether subjects started smoking prior to age 30 and reported smoking at least a pack or more (20+ cigarettes) per day. Never smokers were coded as a zero and used as the reference group in analyses.

### Mortality

Data for mortality follow-up was available via linked mortality files from National Death Index records through 2006 [20]. During analysis, violent, accidental, and HIV deaths were censored as these should not be related to smoking-attributable mortality. Person months of follow-up were provided and converted to years by dividing by twelve. Because participants took part in NHANES III at different times between 1988 and 1994, potential follow-up time was variable, ranging from 12–18 years. To ensure all subjects had the potential to be followed for the same amount of time, 10 year survival was used.

### Physiological Status

In order to examine links between smoking exposure and physiological resiliency, indicators of physiological functioning were selected a priori which, in previous research, have been shown to be affected by cigarette exposure and are also associated with processes related to longevity. For instance, given the inflammatory response to cigarette exposure [22], [23] and the links between chronic inflammation and accelerated-aging [10], we examined measures related to immune activation and inflammation such as CRP, total leukocyte number, lymphocyte number, granulocyte number, and monocyte number.

CRP is protein produced by the liver in response to acute cytokine activation, and as a result is often used as a convenient marker of general systemic inflammation [24]. Measures of CRP were log-transformed in order to improve their distribution. Leukocytes, also known as white blood cells, are immune cells involved in host defense and are composed of various types, including: lymphocytes (T cells, B cells, NK cells), granulocytes (neutrophils, basophils, eosinophils), and monocytes. Overall total leukocytes and its components are increased in response to smoking [25], [26] and have implications for a number of age-related diseases, including but not limited to: cardiovascular disease, stroke, neurodegenerative diseases, cancer, lung disease and diabetes [27]–[31].

In addition, we also examined the associations between smoking, resiliency, and measures of HDL cholesterol and lung function, for which high levels are thought to be beneficial and yet have been shown to be lowered as a result of chronic cigarette exposure [32], [33]. HDL is a lipoprotein which facilitates lipid transport and is protective against cardiovascular disease, neurodegeneration, diabetes, and cancer [34]–[36]. Since the lungs are one of the first areas to interact with the chemicals found in cigarettes, lung function may provide a useful estimate of the amount of tissue damage inflicted by cigarette smoking [8]. Lung function was measured as the ratio between Forced Expiratory Volume at one second and Forced Vital Capacity (FEV1/FVC), which has been shown to correlate with measures of frailty [37], [38].

### Potential Confounders

Age, race/ethnicity, education, sex, and body mass index (BMI) were used as controls in all analyses because these have been related to smoking, physiological outcomes and mortality. Age was top-coded at 90 in the original NHANES data set to protect confidentiality of respondents. This should not affect results since, for the majority of the analysis, persons are classified into four age groups (50–59 years, 60–69 years, 70–79 years, and 80+). Three race/ethnicity categories are included: non-Hispanic whites, non-Hispanic blacks, and Hispanics, most of whom are Mexican Americans. In analyses, Non-Hispanic whites are used as the reference category. Education is measured as years of schooling completed and is included as a continuous variable. Sex was indicated with a dichotomous variable, with females coded as 1. Finally, BMI was calculated as height in meters divided by weight in kilograms squared.

### Statistical Analysis

All analyses were run, using sample weights and controlling for potential confounders including race/ethnicity, education, sex, and BMI. Sample weights are calculated and provided by NHANES. They are used to account for the complex sampling design employed by NHANES. Weights are assigned to each participant in order to represent the number of persons in the U.S. population with given sociodemographic characteristics. As a result, when weights are used in analysis, a sample can be said to be representative of the U.S. population. The association between mortality and smoking, age, and an interaction for age by smoking was modeled using a proportional hazard model with a Gompertz distribution [39]. Based on these results, age-stratified mortality models were used to estimate the hazard of smoking in each age group. These models were first run with the inclusion of all smokers and then rerun, limiting the smoking sample to heavy smokers. This was done to ensure that the proportion of light smokers in the old age group was not driving results. Next, ordinary least squares regression models were used to examine the association between biomarkers and age by smoking interactions. From these models, predicted means for HDL, log CRP, leukocyte number, lymphocyte number, granulocyte number, monocyte number, and FEV1/FVC ratio were estimated and compared between smokers and non-smokers within each age group. Finally, in order to examine whether biomarkers were associated with survival among current and never smokers, proportional hazard models were run testing for associations between biomarkers and mortality in smokers and never smokers, controlling for age, sex, education, race/ethnicity, and BMI.

## Results

### Sociodemographic Characteristics by Age and Smoking Status

Within our population, smoking prevalence was highest for those ages 50–59 (40%) and was lower in each subsequent age group, becoming fairly rare among those 80+ (8%) (Table 1). Overall, differences by sex and socioeconomic status (SES) were consistent with what would be expected—the smoking group included a higher proportion of males and individuals with low education, while at the same time, older cohorts were made up of smaller proportions of males and individuals with low education. Based on these frequencies, SES and sex did not appear to play a greater role in survival for current smokers compared to never smokers. This assumption was tested empirically by examining interactions between 1) sex, smoking, and age category, and 2) education, smoking, and age category using proportional hazard models of mortality, and for both models, interactions were not found to be statistically significant.

**Table 1:** Demographic Characteristics by Age and Smoking Status (N = 4,655).

	50–59 years (N = 1,188)		60–69 years (N = 1,471)		70–79 years (N = 1,075)		80+ years (N = 921)	
	Never Smokers	Current Smokers	Never Smokers	Current Smokers	Never Smokers	Current Smokers	Never Smokers	Current Smokers
**Subjects (%)**	61.9	38.1	65.5	34.5	79.3	20.7	92.1	7.9
**Years Smoking, mean**	--	38.3 (5.0)	--	46.4 (6.3)	--	54.6 (8.2)	--	63.0 (8.8)
**Age Started Smoking**	--	16.4 (4.4)	--	17.8 (5.5)	--	19.4 (7.8)	--	19.7 (7.8)
**Heavy Smoking (%)**	--	74.6	--	66.6	--	55.5	--	41.8
**Female (%)**	71.1	41.3	68.0	51.4	77.8	49.5	78.7	55.2
**White (%)**	81.9	81.6	82.3	81.7	87.4	87.0	88.7	88.5
**Black (%)**	9.3	13.4	9.1	12.1	9.3	9.2	8.3	5.8
**Hispanic (%)**	8.8	5.0	8.6	6.2	3.3	3.8	2.9	5.7
**Education, mean**	12.4 (3.4)	11.5 (3.1)	11.5 (398)	10.8 (3.3)	10.7 (3.7)	10.4 (3.5)	10.0 (3.8)	10.1 (4.1)
**BMI** a **, means**	28.5 (5.8)	26.4 (5.1)	27.9 (5.4)	25.8 (5.4)	27.2 (5.8)	24.7 (4.9)	25.2 (4.6)	22.9 (3.8)
**Died (%)**	4.3	13.6	12.8	36.5	33.9	53.1	72.8	77.0

a BMI: Body Mass Index. All values are run using sample weights

### Age Effects of Smoking on Mortality

A proportional hazard model (Gompertz distribution), controlling for race/ethnicity, education, sex, and BMI was used to examine the association between smoking and mortality for each age group. Overall, we found that while both higher age and smoking were related to an increased risk of mortality, the association between smoking and mortality was significantly reduced in the oldest age group (HR: 0.40; 95% CI: 0.21-0.78) (Table 2).

**Table 2:** Mortality Effects of Smoking and Age, and the Influence of Daily Smoking Quantity.

	Hazard Ratio	95% Confidence Interval
**Female**	0.77	0.66–0.90
**Education**	0.97	0.95–0.99
**Black**	1.28	1.09–1.51
**Hispanic**	0.67	0.45–1.01
**BMI**	1.00	0.99–1.02
**Age (60 years)**	3.06	1.84–5.09
**Age (70 years)**	9.07	5.64–14.60
**Age (80 years)**	29.76	18.70–47.35
**Smoking**	3.01	1.73–5.23
**Age (60) by Smoking**	0.98	0.52–1.85
**Age (70) by Smoking**	0.59	0.32–1.09
**Age (80) by Smoking**	0.40	0.21–0.78

a Proportional Hazard model was run with mortality as the outcome, with person-years of exposure included.

b Overall, 2,393 deaths occurred over a total of 52,144 person-years

Given that significant age by smoking interactions were found for mortality, we used age-stratified proportional hazard models to determine the hazard ratio for current smokers versus never smokers, within each age group. Results showed that the relative mortality risk associated with smoking was extremely high for younger age groups; however, it lessened considerably for older age groups, to the point where smoking no longer significantly contributed to increased mortality risk for subjects who were 80 years of age and older (Table 3). Among subjects ages 50–59, current smokers had an over 4 fold increase in mortality risk compared to never smokers (HR: 4.16; *P*<.001). The risk of mortality from smoking was slightly lower for those ages 60–69, with current smokers being more than 3 times as likely to die as never smokers (HR: 3.36; *P*<.001). For those ages 70–79, current smoking was associated with a 73% increase in the risk of mortality (HR: 1.73; *P*<.001). Nevertheless, among those in the oldest age group, no significant increase in mortality risk was found for current smokers relative to never smokers (HR: 1.31; *P* = .079).

**Table 3:** Hazard Ratios of Current Smoking and Heavy Smoking by age.

	Hazard Ratio (P-value)			
	Ages 50–59	Ages 60–69	Ages 70–79	Ages 80+
**N Deaths**	223	604	698	868
**Person-Years**	16,518	18,379	11,188	6,060
**Current Smoking** a	4.16 (<.001)	3.36 (<.001)	1.73 (<.001)	1.31 (.079)
**Heavy Smoking** a **^,^** b	5.04 (<.001)	3.77 (<.001)	2.50 (<.001)	1.57 (.062)

a Reference group is never smokers

b Heavy smoking defined as smoking uptake prior to age 30 and smoking at least a pack or more (20+ cigarettes) per day. Models were run controlling for sex, race/ethnicity, education, BMI, and age.

Finally, to ensure that lower smoking-related mortality risks at older age weren't resulting from an increased proportion of light smokers or those who started later in life among the 80+ age group, models were rerun including only never smokers and heavy smokers, who we defined as current smokers who began smoking prior to age 30 and reported smoking an average of 20 or more cigarettes per day (Table 3). Similar results were found to those reported above. Overall, the relative risks associated with smoking were highest at younger ages and were no longer significant for subjects ages 80+. Heavy smokers ages 50–59 had a more than 5 fold increase in the risk of mortality compared to never smokers (P<.001). Heavy smokers in their sixties and seventies were approximately 3.8 and 2.5 times as likely to die as never smokers (P<.001), respectively; and finally among those age 80 and above, there was no significant increase in mortality for heavy smokers versus never smokers (P = .062).

### Age Effects of Smoking on Physiological Health

Four independent regression models were used to examine the age-effects of smoking on indicators of physiological health, measured by levels of HDL, log CRP, leukocyte number, lymphocyte number, granulocyte number, monocyte number, and FEV1/FVC ratio (Table 4). Results showed that overall both smoking and age were significantly associated with worse physiological status. However, statistically significant interactions for smoking by age were also found, suggesting that, overall, smokers and non-smokers appeared to have very different age trends, which may be a result of differential mortality selection within the two groups. At younger ages, smoking was related to worse biomarker levels—lower HDL and FEV1/FVC and higher log CRP, leukocyte number, lymphocyte number, granulocyte number, and monocyte number. However, for older subjects, the differences in HDL, CRP, leukocyte number, lymphocyte number, granulocyte number, and monocyte number between smokers and non-smokers were significantly reduced or eliminated (*P*<.05)—suggesting that, at ages 80 and above, current smokers may have similar physiological statuses to never smokers.

**Table 4:** Regression Coefficients of the Association between Current Smoking and Biomarkers.

	FEV1/FVC	HDL	Log CRP	Leukocyte	Monocyte	Lymphocyte	Granulocyte
**Sex (Female = 1)**	0.023***	10.686***	0.100***	−0.208*	−0.035***	0.074	−0.230**
**Education**	0.000	0.232**	−0.006	−0.009	−0.001	−0.007	−0.002
**Black**	0.210***	7.365***	0.173***	−0.996***	−0.067***	0.086*	−1.033***
**Hispanic**	0.220***	−0.802	0.009	0.174	0.028	0.100	0.043
**BMI**	0.003***	−0.822***	0.036***	0.063***	0.005***	0.023***	0.036***
**Age (60–69)**	−0.020***	−0.988	0.047	0.105	0.014	0.025	0.060
**Age (70–79)**	−0.038***	−2.553**	0.092*	0.448***	0.027	−0.033	0.437***
**Age (80+)**	−0.039***	−2.606*	0.187***	0.960***	0.075***	0.027	0.841***
**Smoking** a	−0.075***	−3.785**	0.231***	2.210***	0.140***	0.437***	1.636***
**Age (60–69) by Smoking**	−0.011	2.210	−0.009	−0.524*	−0.029	−0.024	−0.471*
**Age (70–79) by Smoking**	0.002	4.449*	−0.010	−0.849**	−0.058*	−0.125	−0.688**
**Age (80+) by Smoking**	0.012	5.523*	−0.129	−1.538***	−0.089*	−0.344*	−1.102***
**Constant**	0.689	5.049	65.926	0.121	5.049	1.516	3.223
**R-squared**	.203	.172	.088	.149	.081	.042	.157
**N**	4075	4366	4334	4404	4323	4404	4323

* p<0.05; ** p<0.01; *** p<0.001

Results Based on separate OLS Regression Models

a Smoking refers to current smoking

**Figure 1:**
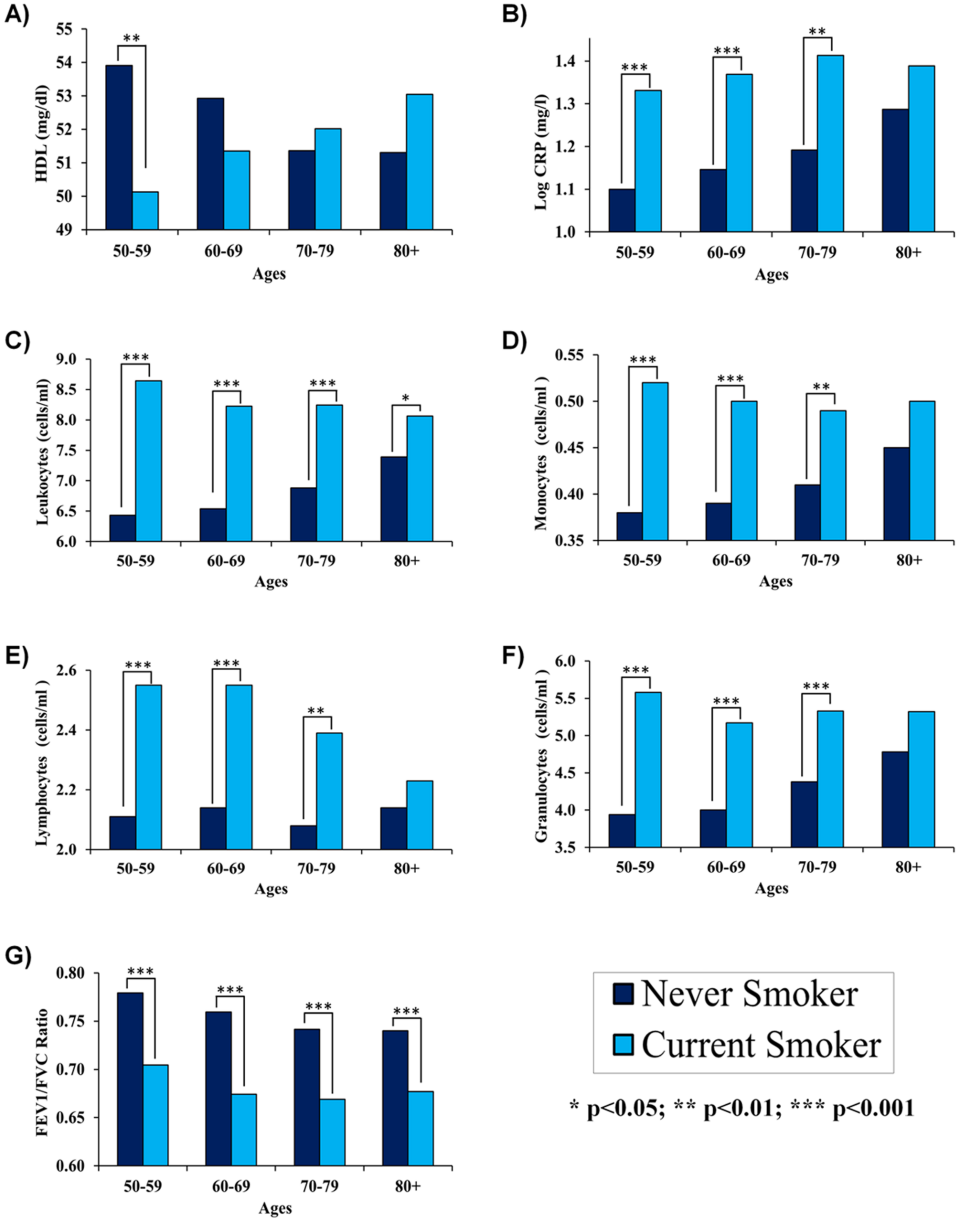
Age Trends in the Association between Smoking and Biomarkers. A cross-over effect was found when comparing HDL by smoking status and age (a) with non-smokers having higher HDL at younger ages, and smokers having higher HDL at older ages. For CRP, leukocyte number, monocyte number, lymphocyte number, and granulocyte number the difference between smokers and non-smokers was largest for subjects in their fifties (b–f). However, these differences appeared to converge with age and were not significantly different for CRP, monocyte number, lymphocyte number, and granulocyte number after age 80. Finally, FEV1/FVC was lower for non-smokers across the age range, and remained statistically significant (g).

From these models, adjusted levels of each marker were calculated for the eight smoking by age groups, controlling for race/ethnicity, education, and sex (Figure 1). These results showed that as the age of the groups increased, differences between smokers and non-smokers were less pronounced or even reversed. For instance, a cross-over effect was found when comparing HDL of current and never smokers over the age range (Figure 1a). At younger ages, never-smokers were found to have significantly higher HDL (*P* = .006)—53.91 mg/dl for never-smokers ages 50–59, compared to only 50.12 for current smokers ages 50–59. However, for each subsequent age group, the difference in HDL by smoking status was smaller, and became no longer significant among those ages 60 and above. Furthermore, there was evidence of a cross-over effect given that for subjects eighty and over, the predicted HDL was higher for current smokers (53.04 mg/dl) than for never smokers. However, this did not reach statistical significance.

When comparing log CRP by smoking, current smokers had higher levels at each age (Figure 1b). Among subjects in their fifties and sixties, current smokers had about 0.23 mg/l (*P*<.001) and 0.22 mg/l (*P*<.001) higher predicted log CRP than never smokers, respectively. For those ages 70–79, log CRP remained significantly higher for smokers—1.41 mg/l compared to 1.19 mg/l for never smokers (*P* = .007). However, for subjects age 80 and over the differences decreased and were no longer significant. For current smokers ages 80 and above, log CRP was 1.39 mg/l, which was only 0.10 mg/l higher (*P* = .319) than log CRP for never smokers in this age range (1.29 mg/l).

Similar patterns were found when examining differences in leukocyte number, lymphocyte number, granulocyte number, and monocyte number (Figure 1c–f). For subjects in their fifties, leukocyte numbers were 2.21×10^3^ cells/µl higher for smokers compared to non-smokers, lymphocyte numbers were 0.437×10^3^ cells/µl higher for smokers compared to non-smokers, granulocyte numbers were 1.64×10^3^ cells/µl higher for smokers compared to non-smokers, and monocyte numbers were 0.139×10^3^ cells/µl higher for smokers compared to non-smokers. However, the differences were smaller for each subsequent age group. When comparing never and current smokers ages 60–69, 70–79, and 80 and above, differences in leukocyte numbers (×10^3^ cells/µl) were 1.69, 1.36 and 0.67, respectively; differences in lymphocyte numbers (×10^3^ cells/µl) were 0.41, 0.31, 0.09, respectively; differences in granulocyte numbers (×10^3^ cells/µl) were 1.17, 0.95, 0.53, respectively; and differences in monocyte numbers (×10^3^ cells/µl) were 0.11, 0.08, and 0.05, respectively. Overall, these differences were significant for ages 50–59, 60–69, and 70–79. However, among those ages 80 and above, differences were only significant for leukocyte number *P* = .04.

Finally, never-smokers had significantly higher FEV1/FVC, regardless of age (Figure 1g). Overall, differences in FEV1/FVC between never and current smokers remained relatively stable for the four age groups, with differences of 0.07% for subjects ages 50–59, 0.09% for subjects 60–69, 0.07% for subjects ages 70–79, and 0.06% for subjects ages 80 and over.

### Associations between Biomarkers and Survival

To determine whether variations in biomarkers, which could be a sign of resiliency, are associated with susceptibility to death, proportional hazard models were run, controlling for age, sex, race/ethnicity, education and BMI, for never smokers and current smokers to estimate the associations between biomarkers and mortality risk within the two groups (Table 5). Levels of log CRP, leukocyte numbers, monocyte numbers, and granulocyte numbers were associated with mortality in both never smokers and current smokers. However, the strength of these associations was larger in the smoking group. A one unit increase in log CRP was associated with 32% increase in mortality risk for current smokers (HR:1.32; *P*<.001), and a 21% increase in mortality risk for never smokers (HR:1.21; *P*<.001). Similarly, one unit increases in Leukocyte, Monocyte, and Granulocyte numbers were significantly associated with 10%, 84%, and 12% increases in mortality risk for current smokers, respectively, and 3%, 47%, and 11% increases in mortality risk for never smokers. Finally, although they were not associated with mortality risks for never smokers, among current smokers, FEV1/FCV was significantly associated with mortality risk (*P*<.001) and Lymphocyte number was marginally associated with mortality risk (*P* = .09).

**Table 5:** Associations between Biomarkers and Mortality for Current and Never Smokers.

	Current Smokers		Never Smokers	
	Hazard Ratio	*P* Value	Hazard Ratio	*P* Value
**FEV1/FVC**	0.04	<.001	0.83	0.7049
**HDL**	0.99	0.6627	0.99	0.0017
**Log CRP**	1.32	<.001	1.21	<.001
**Leukocyte**	1.1	<.001	1.03	0.0057
**Monocyte**	1.84	0.0206	1.47	0.0029
**Lymphocyte**	1.09	0.0922	0.99	0.57
**Granulocyte**	1.12	<.001	1.11	<.001

## Discussion

Based on our results, the risk of death associated with smoking is significantly lower at older ages, to the point where smoking no longer increases mortality for individuals who survive to age 80 and beyond. Furthermore, this does not appear to be a result of cohort differences in smoking habits, as similar patterns are found when comparing only heavy smokers to never smokers. Differences in physiological health by smoking status also converged at older age groups. In younger populations, current smokers had significantly elevated levels of inflammation, immune activation and lower HDL and lung function compared to never smokers. However, at older ages differences between current and never smokers were significantly lower or non-existent. Furthermore, mortality among smokers was strongly related to differences in inflammation and immune activation, as well as lung function.

Given that older subjects had significantly more years of cigarette exposure, one would presume that in a homogenous population, as years of smoking increased, disparities in health between non-smokers and smokers would diverge. However, the increasing similarity between smokers and non-smokers with age, suggests that surviving smokers may represent a distinct sub-population who may possess physiological factors that allow them to either avoid or repair the damage imposed by cigarettes. For instance, compared to shorter-lived smokers, long-lived smokers may exhibit different immunologic responses to biological stressors. We showed that levels of CRP, leukocytes, monocytes, and granulocytes strongly predicted survival, especially among current smokers, which may explain why smokers and non-smokers look more similar as age increases. Furthermore, as expected, smoking was associated with increased immune activation and inflammatory processes for most age groups, as evidenced by the significantly higher CRP, leukocyte, monocyte, lymphocyte, and granulocyte levels among current smokers relative to never smokers. However, long-lived smokers had CRP, monocyte, lymphocyte, and granulocyte levels that were statistically equivalent to those of long-lived persons who had never smoked.

Genetically linked differences in inflammatory and immune responses to stimuli have been reported in the literature [40]. There are a large number of genes involved in the inflammatory pathways, with significant genomic variation. For instance, the +896G+ TLR4 polymorphism was shown to be associated with higher IL-10 levels—an anti-inflammatory cytokine which limits inflammatory signal and response—and lower IL-6—a pro-inflammatory cytokine involved in the recruitment of leukocytes [41]. Additionally, studies have also shown that single nucleotide polymorphisms (SNPs) in -765GC COX-2 are associated with decreased circulating plasma CRP levels [42].

Given that vascular injury from cigarette smoking has been shown to initiate an immunologic response [8], long-lived smokers may have a genetic predisposition that enables them to maintain low levels of inflammation, attenuating their likelihood of accruing additional tissue damage.

Like inflammation, FEV1/FVC levels among smokers were significantly associated with survival. It has been shown that lung injury is often a result of reactive oxygen species (ROS) that cause oxidative damage to proteins, lipids, and DNA [43]. Membrane lipid peroxidation has the potential to increase cellular damage, decreasing lung function and impacting a number of disease states [44]. Additionally, ROS have also been shown to cause apoptosis, stimulate mucus secretion, and disrupt the extracellular matrix and blood vessels [45]. Finally, given the large surface area and blood supply of the lungs, when exposed to exogenous oxidants such as cigarette smoke, tissue may be particularly vulnerable to oxidative stress and damage [46]. As a result, smokers who have innate mechanisms to reduce or offset ROS-induced damage may maintain better lung functioning regardless of cigarette exposure, and given the large differences in survival by FEV1/FVC, lung function may be a useful proxy for resiliency among smokers.

A number of animal models have highlighted the associations between stress resistance and longevity. It has been hypothesized that associations between increased resistance to biological stressors and lifespan extension may be due to stronger antioxidant systems activity. For instance, increased enzymatic antioxidant expression is linked to decreases in damage from ROS and has been shown to increase longevity [47]–[50]. Additionally, superoxide dismutase (SOD) has been shown to act as an initial defense mechanism against damage from ROS, and deletions in SOD genes significantly decrease lifespan in flies, mice, and yeast [51]–[60]. Nevertheless, more work is still needed to understand the role antioxidants play in the aging process.

Given that 1) mortality was not increased for smokers who had survived to age 80 and beyond, 2) smoking was found to have less impact on inflammation for long-lived individuals, and 3) lung function and inflammation were strongly associated with survival among smokers, in moving forward more research is needed to identify factors that allow some smokers to survive to extreme old-age, in spite of sixty or more years of cigarette exposure. In human populations, genetic factors have been estimated to account for approximately 25% of the variation in longevity; however, for those living into their 90 s and 100 s the force of heritability on lifespan is predicted to be even higher [61]. Furthermore, long-lived mutant strains have been identified for a number of specifies, including the nematode Caenorhabditis elegans (C. elegans), Drosophila, and mice [62]–[64], and many of these mutations have been found to be associated with increased levels of stress resistance. Future studies that examine genetic factors such as single nucleotide polymorphisms (SNPs), gene-networks, or gene expression—paying particular attention to processes and pathways involved in inflammation and oxidative stress—may be important for identifying such factors.

There are limitations in the present study that should be acknowledged. The use of cross-sectional biomarker data prevents us from examining changes or trajectories in physiological characteristics of long-lived and short-lived smokers. Also, the small sample size of individuals, particularly older smokers, prevented us from comparing groups at even older ages. Third, data for smoking quantity was based on retrospective self-reports and asked only about current and heaviest smoking levels. As a result, our estimates of smoking quantity may be somewhat biased. Finally, age cohort and gender patterns in smoking history are not random, and therefore hinder our ability to accurately compare between age groups or make estimates or predictions of past or future mortality rates.

Our study is novel in defining a sub-population that may possess high levels of innate physiological resiliency. It presents evidence that long-lived smokers represent a distinct and biologically advantaged group, who are less susceptible to the negative side effects of smoking. Given what we know about smoking and the aging process, the investigation of long-lived smokers provides a natural experiment to examine the ways in which deterministic and stochastic processes interact to impact the rate of aging and the susceptibility to death and disease. In moving forward, more research is needed to facilitate our understanding of the interaction between environmental and genetic mechanisms that influence the degree of degradation with age and to enhance our understanding of factors which influence resiliency and its effect on longevity.
